# Experimental assessment of computationally nominated small molecules targeting the YAP–TEAD interface in Hippo pathway-altered mesothelioma cell models

**DOI:** 10.3389/fonc.2026.1908974

**Published:** 2026-09-14

**Authors:** Glen J. Weiss, Joseph C. Loftus, David W. Mallery, Nhan L. Tran

**Affiliations:** 1 International Genomics Consortium, Phoenix, AZ, United States; 2 Department of Medicine, UMass Chan Medical School, Worcester, MA, United States; 3 Department of Cancer Biology, Mayo Clinic Arizona, Phoenix, AZ, United States; 4 Division of Immunology and Microbiome, Translational Genomics Research Institute, Phoenix, AZ, United States; 5 Department of Integrative Translational Sciences, Beckman Research Institute, City of Hope, Duarte, CA, United States

**Keywords:** drug discovery, Hippo signaling pathway, in silico docking, malignant pleural mesothelioma, negative results, target engagement, virtual screening, YAP/TAZ–TEAD

## Abstract

Aberrant activation of the Hippo–YAP/TAZ–TEAD transcriptional axis is a recurrent feature of multiple solid tumors and contributes to tumor maintenance, lineage plasticity, and therapeutic resistance. Recent medicinal chemistry has shown that TEAD proteins are druggable, but the rate at which in silico screening nominates compounds that prove functionally active in cells remains poorly defined, and negative validation results are rarely reported. Here we describe an experimental assessment of a focused set of computationally nominated candidate compounds, selected from a structure-guided virtual screen of the YAP–TEAD interface across approximately 917,000 library compounds and three predicted binding sites. Candidates were evaluated using long-term (144 hour) CellTiter-Glo proliferation assays in three Hippo-altered malignant pleural mesothelioma cell lines (MSTO-211H, NCI-H2052, NCI-H226), with the validated TEAD inhibitor K-975 and cisplatin as positive controls, and an exploratory TEAD-responsive luciferase reporter in MCF7 cells. K-975 produced dose-dependent antiproliferative activity in MSTO-211H and NCI-H2052, with reduced sensitivity in NCI-H226, while cisplatin reduced viability in all lines. In contrast, the computationally nominated compounds, including AG-690/36549045, AN-655/40856217, and additional library members, did not produce measurable antiproliferative activity at the concentrations tested. In the exploratory reporter, apparent suppression of TEAD activity consistently co-occurred with loss of cell viability, a pattern consistent with cytotoxicity rather than selective transcriptional inhibition; no TEAD-active reference compound was co-tested within the reporter panel, and no direct YAP–TEAD biochemical or target-engagement assay was performed. These results describe a case in which computational nomination at the YAP–TEAD interface showed limited concordance with cell-based activity and provide a transparent account of negative findings to inform expectations for early-stage discovery targeting Hippo-dependent transcriptional programs.

## Introduction

The Hippo signaling pathway is a highly conserved regulatory network that controls organ size, tissue homeostasis, and regeneration by modulating the activity of the transcriptional co-activators Yes-associated protein (YAP) and transcriptional co-activator with PDZ-binding motif (TAZ) (1–3). In canonical Hippo signaling, upstream kinases including MST1/2 and LATS1/2 phosphorylate YAP and TAZ, leading to cytoplasmic sequestration and proteasomal degradation (1–4). When Hippo signaling is disrupted, YAP and TAZ accumulate in the nucleus, where they associate with TEA domain transcription factors (TEAD1–4) to drive gene expression programs that promote proliferation, survival, epithelial–mesenchymal plasticity, and resistance to therapy (2–7).

Dysregulation of the Hippo–YAP/TAZ–TEAD axis is a feature of multiple malignancies (3–13). In malignant pleural mesothelioma, loss-of-function alterations in NF2 or LATS1/2 occur at high frequency and confer strong dependence on YAP/TAZ-mediated transcriptional output (14–17). Elevated YAP/TAZ activity has also been implicated in non-small cell lung cancer, colorectal cancer, pancreatic ductal adenocarcinoma, renal cell carcinoma, and hepatocellular carcinoma, either through genetic alterations or via pathway crosstalk with oncogenic signaling cascades such as KRAS, EGFR, and MAPK (3, 7–9, 11, 13, 18, 19). Beyond tumor initiation, YAP/TAZ signaling contributes to adaptive resistance to targeted therapies and cytotoxic stress, further reinforcing its appeal as a therapeutic target (7, 8, 10, 13, 19–22).

Although transcription factor complexes were long considered undruggable, recent structural and medicinal chemistry advances have demonstrated that TEAD proteins contain a conserved palmitoylation pocket that can be exploited pharmacologically (18, 21–24). Both covalent and non-covalent TEAD inhibitors have shown robust preclinical activity in Hippo-altered tumor models, validating TEAD as a druggable node within the Hippo pathway (22, 24).

In parallel with these advances, computational and in silico screening approaches have increasingly been applied to identify candidate YAP/TEAD pathway modulators from large chemical libraries (13, 21, 23). Structure-based and ligand-based virtual screening has been applied to the YAP–TEAD interface and the TEAD palmitate pocket in numerous published campaigns over the past decade, yet only a small minority of computationally nominated chemotypes have advanced to validated cellular activity, and the clinical-stage TEAD inhibitors reported to date have arisen predominantly from biochemical and structure-based medicinal-chemistry programs rather than from in silico docking alone. This gap between the volume of nominated compounds and the number with confirmed cellular activity motivated the present study. While such approaches offer scalability, the proportion of nominated compounds that prove functionally active in cells remains poorly defined. Phenotypic effects observed *in vitro* may also arise indirectly through stress responses, pleiotropic signaling, or convergence on YAP/TAZ-dependent survival programs rather than through direct modulation of TEAD activity. Negative validation results are seldom reported, which contributes to publication bias and obscures the true difficulty of discovering functional Hippo pathway modulators.

Here we present an experimental assessment of a focused set of computationally nominated candidate compounds, evaluated alongside a validated TEAD inhibitor used as a positive control. Using long-term CellTiter-Glo proliferation assays in three Hippo-altered mesothelioma cell lines and an exploratory TEAD-responsive reporter, we sought to (i) confirm assay sensitivity and biological relevance using a reference inhibitor, (ii) determine whether the nominated compounds produced measurable antiproliferative activity, and (iii) describe, transparently, the concordance between computational nomination and cell-based activity. By reporting negative as well as positive findings, this study offers a realistic reference point for discovery efforts targeting the Hippo–YAP/TAZ–TEAD axis.

## Materials and methods

### Structure-guided virtual screening and molecular modeling of the YAP–TEAD complex

Structure-guided virtual screening and molecular modeling were conducted to identify small-molecule candidates predicted to bind functionally relevant interfaces within the YAP–TEAD transcriptional complex. The objective of this computational campaign was to nominate candidate compounds for experimental testing by targeting sites implicated in the physical association of YAP and TEAD and in the regulation of TEAD transcriptional activity. Computational predictions were used solely for compound prioritization and did not constitute evidence of direct biochemical inhibition.

Structural templates were derived from the crystal structure of the human YAP–TEAD complex deposited in the Protein Data Bank (PDB ID: 3KYS), which contains TEAD1 and YAP1 chains resolved in complex. Missing residues and loops were reconstructed prior to docking, and residue numbering was standardized according to UniProt sequence annotations. Protein preparation included the addition of hydrogen atoms, assignment of bond orders, optimization of protonation states at physiological pH (7.4), and correction of missing side chains using established protein preparation workflows (25, 26).

Three structurally and functionally relevant binding regions within the YAP–TEAD complex were selected for docking based on prior structural and biochemical studies: the alpha-helix interface site, corresponding to the YAP alpha-helix that mediates TEAD binding; the TEAD Cys359 site, a pocket adjacent to the palmitoylation site implicated in TEAD stability and transcriptional activity; and the YAP Phe95 site, a region reported to contribute to YAP–TEAD interaction dynamics. Grid boxes with dimensions of approximately 22.7 × 22.7 × 22.7 Å were centered on each site, with a grid spacing of 0.375 Å and docking exhaustiveness set to 8 (25, 27, 28).

Small-molecule libraries from Otava, Specs, and ChemDiv were used as docking inputs, comprising approximately 917,000 unique compounds after accounting for protonation states and stereoisomers. Ligand preparation used standard AutoDock tools, including assignment of Gasteiger charges and generation of Cartesian coordinates. Docking was carried out using AutoDock Vina, with each compound docked independently into all three sites, for approximately 2.75 million docking calculations in total. Docked poses were ranked by predicted binding affinity and interaction geometry (29).

Top-scoring compounds from each site were subjected to short molecular dynamics (MD) simulations (1 ns) to assess binding stability, and average binding free energies were estimated using the MM/GBSA method from sampled trajectories (30, 31). A subset of compounds with favorable MM/GBSA profiles was further evaluated with long-timescale MD simulations (100 ns), and compounds maintaining stable binding modes were prioritized for experimental evaluation. The computational pipeline was designed to predict binding affinity to YAP–TEAD interfaces and was not configured to predict cellular permeability or pharmacokinetic properties. All molecular modeling, docking, MD simulations, and computational analyses were performed by Istanbul MedChem Health Products Ltd. Co. (Istanbul, Turkey) (29–31).

### Compound selection and nomenclature

Candidate compounds were selected from the computational screen described above on the basis of predicted binding scores, MM/GBSA estimates, MD stability, chemical diversity, and commercial availability for experimental testing. The following compounds were evaluated: K-975, a validated TEAD inhibitor (32), included as a positive control; tiospirone, a computationally nominated candidate compound; AG-690/36549045, a computationally nominated candidate compound; and AN-655/40856217, a computationally nominated candidate compound. Additional computationally nominated library members, including montelukast, were evaluated in the exploratory TEAD reporter described below and are summarized in **Supplementary Table 1**. Compound identities, vendor sources are summarized in **Table 1**.

**Table 1:** Identity, source, and screening classification of compounds evaluated in this study.

Compound	Vendor	Library/catalog ID	MW (Da)	Role in study	Predicted target site
K-975	MedChemExpress	HY-138565	534.6	Reference compound	TEAD palmitate pocket
AG-690/36549045	Specs	AG-690/36549045	433.38	Computationally nominated candidate	TEAD Cys359 pocket
AN-655/40856217	Specs	AN-655/40856217	448.47	Computationally nominated candidate	YAP α-helix interface
Tiospirone	Sigma-Aldrich	T-127	465.6	Computationally nominated candidate	YAP Phe95 site
Cisplatin	Sigma-Aldrich	P4394	300.0	Off-pathway cytotoxicity positive control	DNA damage (off-pathway)

MW, molecular weight. Predicted target site refers to the binding region within the YAP–TEAD complex used during in silico nomination and does not constitute evidence of direct biochemical engagement.

### Cell lines and culture conditions

Human malignant pleural mesothelioma cell lines MSTO-211H (LATS1/2-deficient), NCI-H2052 (NF2-deficient), and NCI-H226 (NF2-deficient) were obtained from authenticated commercial sources and selected to represent Hippo-altered cellular contexts with differential dependence on YAP/TAZ-mediated transcriptional output. Cells were cultured in RPMI-1640 medium supplemented with 10% fetal bovine serum (FBS) and maintained at 37 °C in a humidified incubator with 5% CO_2_. Cell line authentication and mycoplasma testing were conducted by the testing facility prior to initiation of experiments. This study did not involve human participants, human-derived tissues, or animal subjects, and therefore did not require ethics approval.

### Long-term proliferation assays

Cell proliferation was assessed using a luminescent ATP-based CellTiter-Glo (CTG) assay (Promega, Madison, WI, USA), which quantifies intracellular ATP as a surrogate for metabolically active, viable cells. Cells were seeded into white, flat-bottom 96-well plates at 3,000 cells per well and allowed to adhere overnight. The following day, culture medium was replaced with RPMI-1640 containing 1% FBS for a 4-hour equilibration period prior to compound treatment. Test compounds were added as serial dilutions from 10× stock solutions, with a final DMSO concentration of 0.1% (v/v) across all wells, and cells were incubated with compounds for 144 hours without medium exchange. A vehicle-only (0.1% DMSO, no compound) condition was included on every plate as the 100% viability reference. At the endpoint, CTG reagent was added at a 1:1 ratio with culture medium, plates were shaken for 5 minutes to induce cell lysis and incubated at room temperature for 20 minutes, and luminescence was recorded using a multimode microplate reader. Relative viability was calculated by normalization to vehicle-treated controls, and dose-response curves were fitted by nonlinear regression to estimate IC_50_ values where applicable. Each concentration was tested in technical triplicate, and assays were performed in two independent runs. Cisplatin (Sigma-Aldrich, St. Louis, MO, USA) was included as an off-pathway cytotoxicity positive control across the same concentration range. Proliferation assays were conducted by Kyinno Biotechnology Co., Ltd. (Beijing, China). Raw assay outputs supporting these analyses are deposited at the Open Science Framework (33).

### Annexin V apoptosis assay

Apoptosis was assessed using the RealTime-Glo Annexin V Apoptosis and Necrosis Assay (Promega), a live-cell, non-lytic, real-time (kinetic) assay in which phosphatidylserine externalization on the outer plasma membrane leaflet is detected through annexin V binding and reported as a luminescent signal. NCI-H226 cells were seeded in 96-well plates and treated with tiospirone or cisplatin alongside vehicle-treated controls in the presence of the assay detection reagents. Luminescence was recorded on a multimode microplate reader at defined intervals across a 2 to 72 hour window (2, 6, 18, 24, 48, and 72 hours). The annexin V luminescent signal at each interval was expressed relative to vehicle-treated controls, and apoptosis kinetics were classified according to the definitions given in the **Table 2** footnote. Each condition was tested in technical triplicate within a representative experiment. Apoptosis assays were conducted by Kyinno Biotechnology Co., Ltd. (Beijing, China).

**Table 2:** Single-agent RealTime-Glo Annexin V apoptosis summary for tiospirone, and cisplatin in NCI-H226.

Treatment	Treatment context	Cell model(s)	Apoptosis induction	Kinetic profile
Tiospirone	Single agent	NCI-H226	No	No early induction
Cisplatin	Single agent (positive control)	NCI-H226	Yes	Early/progressive

Apoptosis assessed by the RealTime-Glo Annexin V luminescent assay (Promega); annexin V binding is reported as a luminescent signal. Kinetic definitions: Early: Annexin V signal ≥2-fold above vehicle within 6 h. Delayed (24–36 h): divergence from vehicle between 24–36 h. Progressive: sustained increase through 72 h. No early induction: no reproducible increase relative to vehicle over the 2 to 72 h interval. Each condition was tested in technical triplicate within a representative experiment.

### Exploratory TEAD-responsive reporter assay

To explore whether computationally nominated compounds suppressed YAP/TAZ–TEAD-driven transcription, MCF7 cells stably expressing a Hippo pathway TEAD-responsive luciferase reporter were seeded into 96-well plates and the reporter system was activated by addition of okadaic acid (0.5 μM). Compounds were tested in triplicate across a five-point concentration range (10 nM to 100 μM) with a matched DMSO vehicle, and luciferase reporter signal and cell viability were measured in parallel at 24 and 48 hours. Reporter signal was normalized to vehicle-treated controls. Compounds were considered to show selective reporter activity only if they suppressed reporter output without a concurrent reduction in cell viability. This assay was exploratory: it was performed in MCF7 cells rather than in the mesothelioma proliferation models, it did not include a TEAD-active reference compound within the same reporter panel, and it was not designed to establish direct pathway engagement. Reporter assays were conducted by PROGEN Biyoteknoloji Ltd. Şti (Istanbul, Turkey). Reporter data were not used to support any conclusion regarding direct YAP–TEAD engagement (18, 21).

### Data analysis

Data are presented as mean ± standard error of the mean (SEM) of technical replicates from representative experiments unless otherwise stated. Dose-response curves were fit using four-parameter nonlinear regression in GraphPad Prism. IC_50_ values are reported as approximate values where curve fits were well constrained. Given the descriptive scope of the study and the limited number of candidate compounds, qualitative consistency across cell lines and assay runs was emphasized rather than formal hypothesis testing.

### Use of generative AI

Generative AI tools were used to assist with manuscript organization, language refinement, and formatting. Specifically, ChatGPT (GPT-4o; OpenAI, San Francisco, CA, USA) and Claude (claude-sonnet-4.6; Anthropic, San Francisco, CA, USA) were used for these purposes. The authors had full access to the primary experimental data and are solely responsible for data interpretation, scientific conclusions, and the final content of the manuscript. All AI-assisted text was reviewed by the authors for factual accuracy.

## Results

### Benchmarking assay sensitivity with a validated TEAD inhibitor

To establish assay sensitivity and confirm biological relevance in Hippo pathway-altered contexts, we first evaluated the response of the three malignant pleural mesothelioma cell lines to K-975, a validated small-molecule inhibitor of YAP/TEAD transcriptional activity used here as a reference compound. In long-term (144 hour) CellTiter-Glo proliferation assays, K-975 produced dose-dependent antiproliferative activity in MSTO-211H and NCI-H2052 cells, with IC_50_ values in the low-nanomolar range (**Table 3**; representative curves in **Figure 1**). NCI-H226 cells showed markedly reduced sensitivity despite NF2 alteration, indicating context-dependent reliance on YAP/TAZ-driven transcriptional programs across these Hippo-altered models. Cisplatin reduced viability in all three lines (**Table 3**), confirming that the assay detected cytotoxic responses.

**Table 3:** Long-term CellTiter-Glo proliferation outcomes for K-975, cisplatin, and computationally nominated candidate compounds in Hippo-altered mesothelioma cell lines.

Compound	Cell line	Hippo pathway alteration	Approx. IC_50_ (nM)*	Antiproliferative activity
K-975	MSTO-211H	LATS1/2-deficient	≈45	Robust
K-975	NCI-H2052	NF2-deficient	≈85	Robust
K-975	NCI-H226	NF2-deficient	>2,000	Reduced sensitivity
AG-690/36549045	MSTO-211H	LATS1/2-deficient	>2,000	None detected
AG-690/36549045	NCI-H2052	NF2-deficient	>2,000	None detected
AG-690/36549045	NCI-H226	NF2-deficient	>2,000	None detected
AN-655/40856217	MSTO-211H	LATS1/2-deficient	>2,000	None detected
AN-655/40856217	NCI-H2052	NF2-deficient	>2,000	None detected
AN-655/40856217	NCI-H226	NF2-deficient	>2,000	None detected

*Approximate IC_50_ values estimated from four-parameter nonlinear regression of representative dose-response curves; values >2,000 nM denote no measurable activity within the tested concentration range. K-975 is the on-pathway reference inhibitor; cisplatin is an off-pathway cytotoxicity control. Values represent qualitative summaries from two independent assay runs with technical triplicates per concentration. For the nominated compounds, no antiproliferative activity was detected up to the highest concentration tested (2 µM; 2,000 nM). Cisplatin was included as a single-concentration positive control (100 µM) and was not titrated, so no IC_50_ was determined; at 100 µM it reduced viability to approximately 1 to 4% of vehicle across all three lines (MSTO-211H ~1%, NCI-H226 ~3%, NCI-H2052 ~4%).

**Figure 1:**
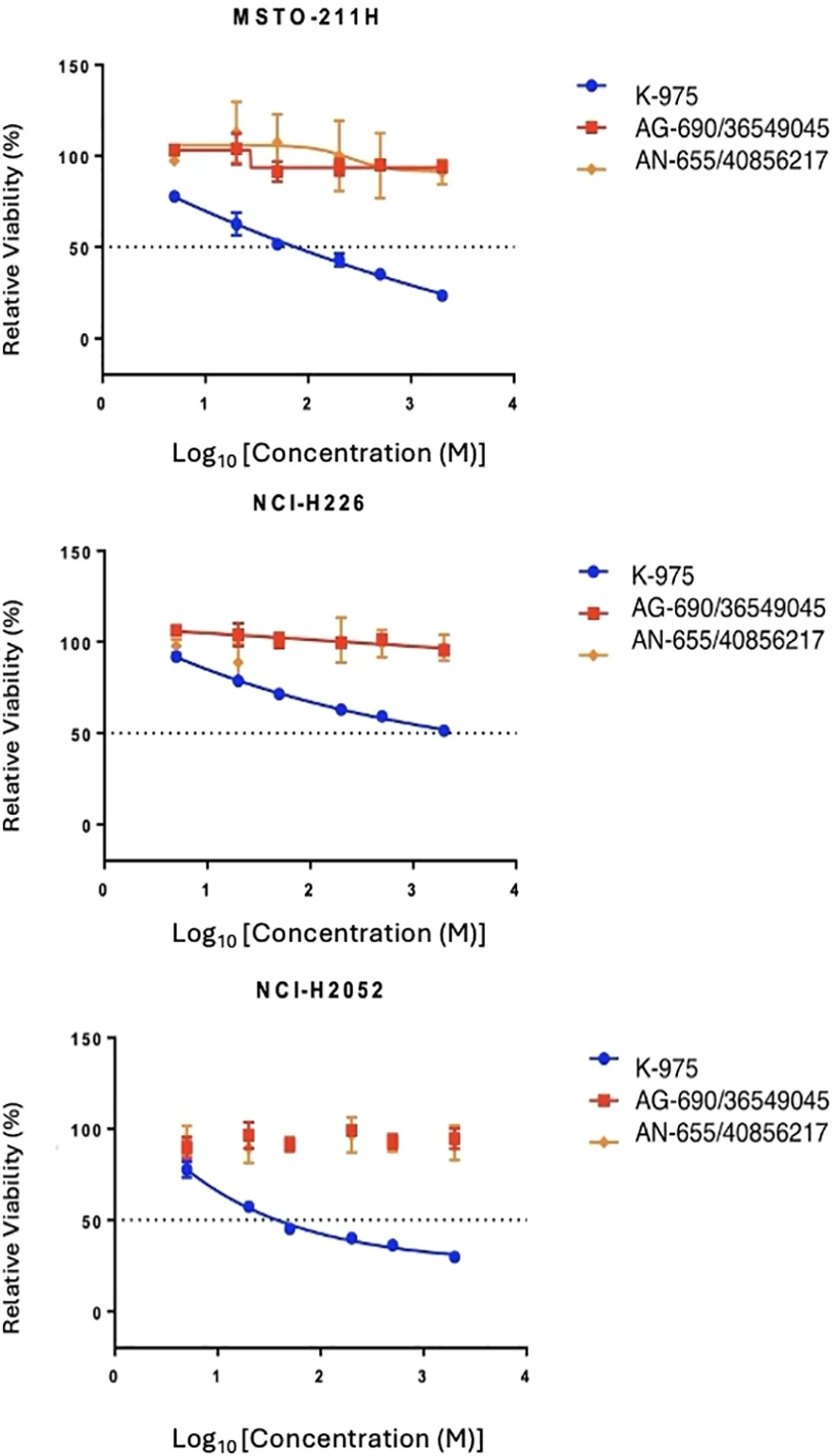
Long-term (144 hour) CellTiter-Glo dose-response curves for K-975, AG-690/36549045, and AN-655/40856217 in MSTO-211H, NCI-H2052, and NCI-H226 mesothelioma cells. Relative viability is normalized to vehicle-treated (0.1% DMSO) controls, shown as the 100% baseline. K-975 produced dose-dependent reductions in relative viability in MSTO-211H and NCI-H2052, with reduced sensitivity in NCI-H226, whereas AG-690/36549045 and AN-655/40856217 produced essentially flat response profiles. Concentrations are expressed in nM. Points represent mean ± SEM of technical triplicates from a representative experiment; assays were performed in two independent runs.

The K-975 sensitivity profile was reproducible across the independent assay runs performed and was consistent with previously reported dependencies on YAP/TEAD signaling in these models (**Table 2**; see also refs. 16, 17, 28). The extended assay duration allowed discrimination between transient cytostatic effects and sustained suppression of proliferation. These results provided a benchmark against which the computationally nominated candidate compounds could be evaluated.

### Computationally nominated candidates AG-690/36549045 and AN-655/40856217 lack antiproliferative activity

Two structurally distinct compounds nominated through the structure-guided virtual screen, AG-690/36549045 and AN-655/40856217, were evaluated across the same mesothelioma cell line panel. Under identical 144-hour continuous exposure conditions, neither compound produced sub-micromolar growth inhibition. Relative to the vehicle-normalized 100% baseline, dose-response curves remained essentially flat across the tested concentration ranges (**Figure 1**), and IC_50_ values exceeded the highest tested concentration in all models (**Table 3**). These findings were reproducible across the independent assay runs performed, under conditions that reliably detected robust pathway-dependent growth inhibition by the positive control. Because activity emerging only at cytotoxic concentrations cannot be distinguished from nonspecific toxicity, and because the exploratory reporter panel tested the same class of compounds to 100 µM and observed only viability-coupled signal loss (see below), the flat proliferation responses are best interpreted as an absence of selective antiproliferative activity rather than as a failure to reach an active concentration. Because aqueous solubility, permeability, and intracellular exposure were not measured, limited cellular uptake cannot be excluded as a contributing factor to the absence of activity.

### Tiospirone shows no measurable single-agent antiproliferative activity

Tiospirone, nominated through the virtual screen as a putative YAP/TEAD pathway modulator, was evaluated in the exploratory MCF7 TEAD-responsive reporter and in the single-agent apoptosis assay rather than in the mesothelioma proliferation panel. Tiospirone did not selectively suppress TEAD reporter activity; apparent reporter suppression co-occurred with loss of cell viability (**Supplementary Table 1**; **Supplementary Figure 1**), and tiospirone did not induce apoptosis (**Table 2**). These findings are consistent with the absence of selective activity observed for the other nominated compounds and argue against classification as a functional TEAD inhibitor in these models.

### A broader set of nominated compounds shows no selective activity

To assess the nominated compounds more broadly, a panel of computationally nominated library members was tested in the exploratory MCF7 TEAD-responsive reporter; the compounds evaluated and their outcomes are summarized in **Supplementary Table 1**, and complete per-compound raw reporter and viability traces are deposited at the Open Science Framework (33). Representative dose-response outputs are shown for montelukast and tiospirone (**Supplementary Figure 1**). Several compounds reduced reporter luminescence at higher concentrations; however, in the majority of cases the reduction in reporter signal co-occurred with a concurrent loss of cell viability, a pattern consistent with cytotoxicity rather than selective inhibition of TEAD-dependent transcription. For montelukast and tiospirone, apparent reporter suppression tracked with parallel declines in cell viability at both 24 and 48 hours (**Supplementary Figure 1**), so that the fitted reporter IC_50_ values reflect viability-coupled signal loss rather than selective transcriptional inhibition (**Supplementary Table 1**). Curve fits for several apparent reporter responses were of low quality, and no compound produced clear suppression of reporter activity in the absence of viability loss. Because no TEAD-active reference compound was co-tested within the reporter panel and the reporter was run in MCF7 cells rather than in the mesothelioma proliferation models, the reporter data were treated as exploratory and were not used to infer direct pathway engagement (**Supplementary Figure 1**; **Supplementary Table 1**).

None of the compounds that produced apparent reporter signal changes translated into measurable antiproliferative activity in the Hippo-altered mesothelioma lines. Across the compounds advanced to proliferation testing and the broader reporter panel, the computationally nominated candidates did not demonstrate reproducible, viability-independent activity and were therefore classified as inactive under the conditions tested (**Table 2**; **Supplementary Table 1**).

## Discussion

This study provides an experimental assessment of computationally nominated candidate compounds targeting the YAP–TEAD interface and describes the concordance between computational nomination and cell-based activity in Hippo-altered mesothelioma models. By benchmarking assay performance with a validated TEAD inhibitor, we confirmed dose-dependent antiproliferative sensitivity in MSTO-211H and NCI-H2052 and reduced sensitivity in NCI-H226. The heterogeneous responses across NF2- or LATS-altered cell lines are consistent with prior reports of context-dependent reliance on YAP/TAZ-driven transcriptional output in malignant pleural mesothelioma (14, 16, 17).

In contrast, the compounds nominated through the structure-guided virtual screen, including AG-690/36549045 and AN-655/40856217 did not produce measurable antiproliferative activity despite prolonged exposure under conditions that reliably detected the activity of the positive control. These observations illustrate the limited concordance that can occur between docking and MM/GBSA-based nomination and cell-based functional activity when nomination is not coupled to early experimental validation. Predicted binding to TEAD interfaces did not translate into transcriptional suppression or phenotypic dependency in these models, consistent with limitations noted in prior efforts to identify YAP–TEAD inhibitors (26, 34). Computational predictions are best interpreted as prioritization tools rather than as evidence of biological activity.

The central limitation of this study is the absence of a direct target-engagement assay. Because nomination was predicated on predicted binding at the YAP–TEAD interface, the first experimental test of that prediction should be a biophysical or cellular engagement measurement, for example surface plasmon resonance, isothermal titration calorimetry, a cellular thermal shift or NanoBRET target-engagement assay, or a TEAD auto-palmitoylation assay. We did not perform such an assay here; consequently, the present data bound only the phenotypic question, and we draw no inference about physical engagement. Establishing engagement is the first step we identify for any future prioritization of these or related nominated chemotypes. Closely coupled to engagement is compound delivery: physicochemical properties such as solubility and passive membrane permeability, which were not directly measured here, may further constrain translation of in silico binding predictions into cellular activity, particularly for higher molecular weight compounds. Because the computational pipeline was not designed to predict cellular permeability, the absence of cellular activity cannot be attributed solely to deficiencies in the binding model. Future workflows should incorporate early permeability and uptake assays, for example PAMPA, Caco-2, or intracellular compound quantification, alongside an engagement assay, so that delivery failure and engagement failure can be separated before compounds are committed to phenotypic screening.

The exploratory TEAD reporter merits specific caution. Apparent suppression of reporter activity consistently co-occurred with loss of cell viability, indicating that reduced luminescence most plausibly reflects fewer viable, signal-producing cells rather than selective inhibition of TEAD-dependent transcription. Two features of the reporter design limit its interpretability: no TEAD-active reference compound was tested within the same reporter panel to anchor the dynamic range, and the reporter was run in MCF7 cells rather than in the mesothelioma proliferation models. For these reasons we did not use the reporter to infer pathway engagement, and we report it only as an exploratory readout. A reporter configuration that includes a validated TEAD inhibitor on the same plate and that normalizes reporter signal to a parallel viability measurement would be required before any reporter-based claim of selective TEAD inhibition could be made.

Several further limitations should be emphasized. First, no direct biochemical or cellular YAP–TEAD target-engagement assay was performed, so we cannot state whether the nominated compounds bound TEAD or disrupted the YAP–TEAD interaction; the data establish only the absence of antiproliferative activity in the models tested. Second, the TEAD reporter data are exploratory and confounded by cytotoxicity, as described above. Third, the cell-line panel was limited to Hippo-altered mesothelioma. We selected this context deliberately, because NF2- and LATS-altered malignant pleural mesothelioma represents one of the most stringent and best-validated settings of YAP/TAZ dependence and is a standard test-bed for TEAD inhibitor validation; a compound inactive in this context is unlikely to be a strong selective TEAD inhibitor, although lineage-specific activity elsewhere cannot be excluded and the findings may not generalize to other YAP/TAZ-dependent contexts. Fourth, only a small number of computationally nominated compounds were advanced to long-term proliferation testing, and physicochemical and pharmacokinetic properties were not measured. These constraints bound the conclusions to a description of the activity observed under the specific conditions tested.

A notable aspect of this work is the explicit reporting of negative and null results, which remain underrepresented in the literature despite their importance for translational research. Publication bias toward positive results obscures the true difficulty of identifying functional Hippo pathway modulators, particularly given the complex biology of transcriptional co-activators and protein-protein interactions (21, 34). By documenting both the validated positive control and the failure of nominated candidates to show activity, these data provide a realistic reference point for the predictive limits of in silico screening at the YAP–TEAD interface.

Future studies will be required to determine whether any computationally nominated compound engages TEAD or the YAP–TEAD interface directly. As a priority, this work should extend to Hippo-altered models from additional lineages and should incorporate direct transcriptional readouts of TEAD activity, in particular quantitative PCR of canonical target genes (CTGF/CCN2, CYR61/CCN1, and ANKRD1), which would establish whether a nominated compound modulates TEAD-dependent transcription independently of effects on viability. Coupling these readouts to a viability-normalized, reference-anchored reporter, together with direct target-engagement assays such as co-immunoprecipitation, split-luciferase interaction assays, or TEAD autopalmitoylation assays, will be essential to resolve these questions. More broadly, incorporating early functional and pathway-specific readouts into screening pipelines may help focus chemistry efforts on candidates with genuine biological leverage over the Hippo–YAP/TAZ–TEAD axis.

## Conclusions

In a structure-guided virtual screen of the YAP–TEAD interface followed by cell-based assessment, computationally nominated candidate compounds did not reproduce the antiproliferative activity of a validated TEAD inhibitor in Hippo-altered mesothelioma models, and apparent activity in an exploratory TEAD reporter was confounded by cytotoxicity. These transparent negative results illustrate the limited concordance that can occur between computational nomination and cellular activity, and support coupling in silico nomination to early functional and target-engagement assays in future Hippo pathway discovery efforts.

## Data Availability

The datasets presented in this study are publicly available. Raw CellTiter-Glo viability, Annexin V apoptosis, and TEAD reporter assay data, including per-compound reporter and viability traces for the full reporter panel, are deposited at the Open Science Framework (OSF) and can be accessed at https://doi.org/10.17605/OSF.IO/ZTH9S under a Creative Commons Attribution 4.0 International (CC-BY 4.0) license (33).

## Supplementary material

Supplementary Figure 1 Exploratory MCF7 TEAD-responsive luciferase reporter outputs for representative computationally nominated compounds (montelukast and tiospirone), showing reporter luminescence and parallel cell viability at 24 and 48 hours across concentration. Reductions in reporter signal co-occurred with reductions in cell viability. Fitted IC_50_ values for apparent reporter suppression are indicated and reflect viability-coupled signal loss. LFU, luciferase luminescence units; RFU, relative fluorescence units (viability). Upper panels: dual-axis plots of reporter luminescence (left y-axis) and cell viability (right y-axis). Lower panels: fitted dose-response curves for reporter luminescence with IC_50_ annotations. Compounds were tested in technical triplicate across a five-point concentration range (10 nM to 100 µM).
