# Supplementary material for: Experimental assessment of computationally nominated small molecules targeting the YAP–TEAD interface in Hippo pathway-altered mesothelioma cell models
Source: Front Oncol. 2026 Sep 14;16:1908974. doi: 10.3389/fonc.2026.1908974 (PMC13616692; doi:10.3389/fonc.2026.1908974)
Supplement: Supplementary file 1 [file DataSheet1.pdf]

Montelukast

24h treatment

48h treatment

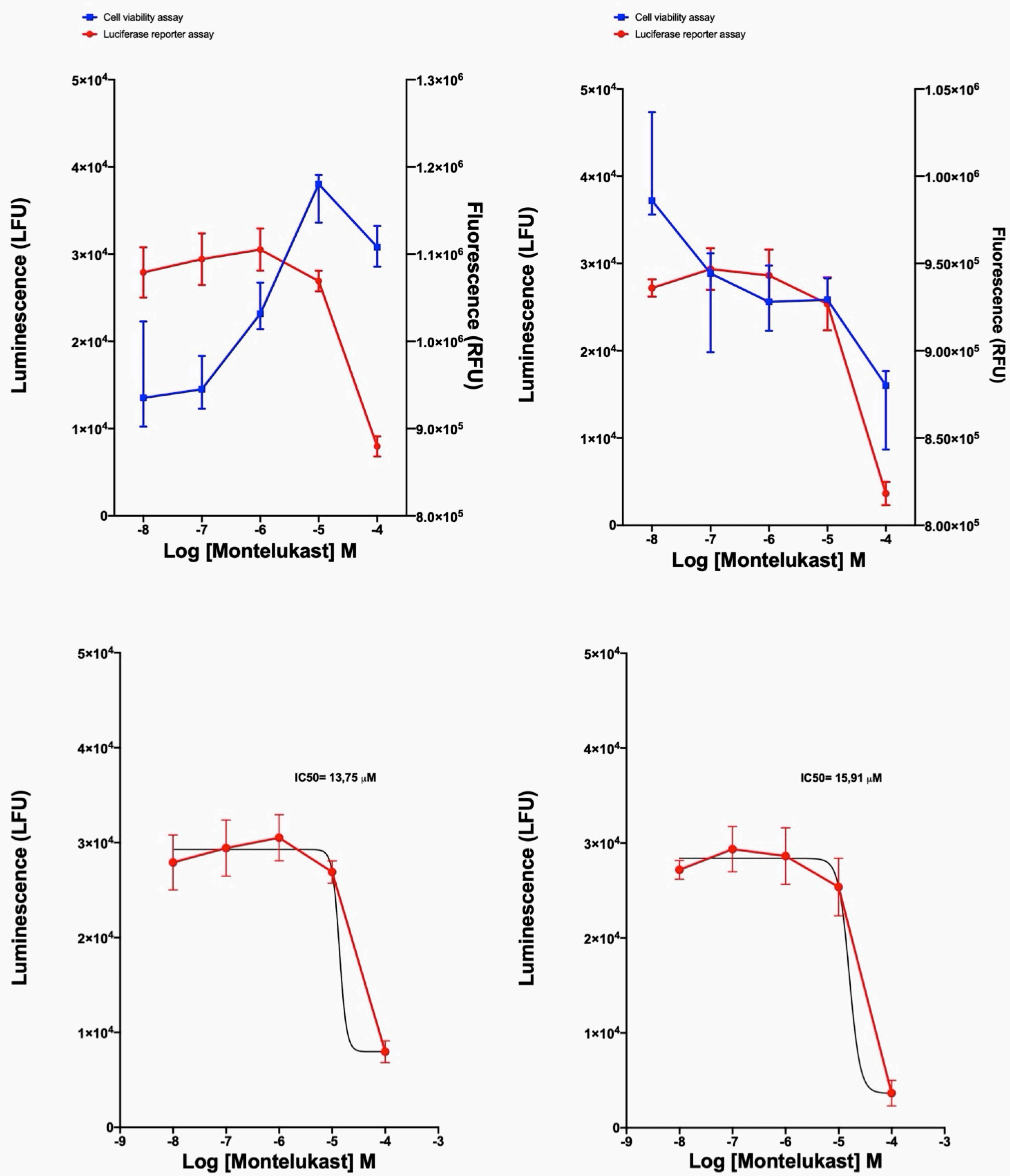

Tiospirone

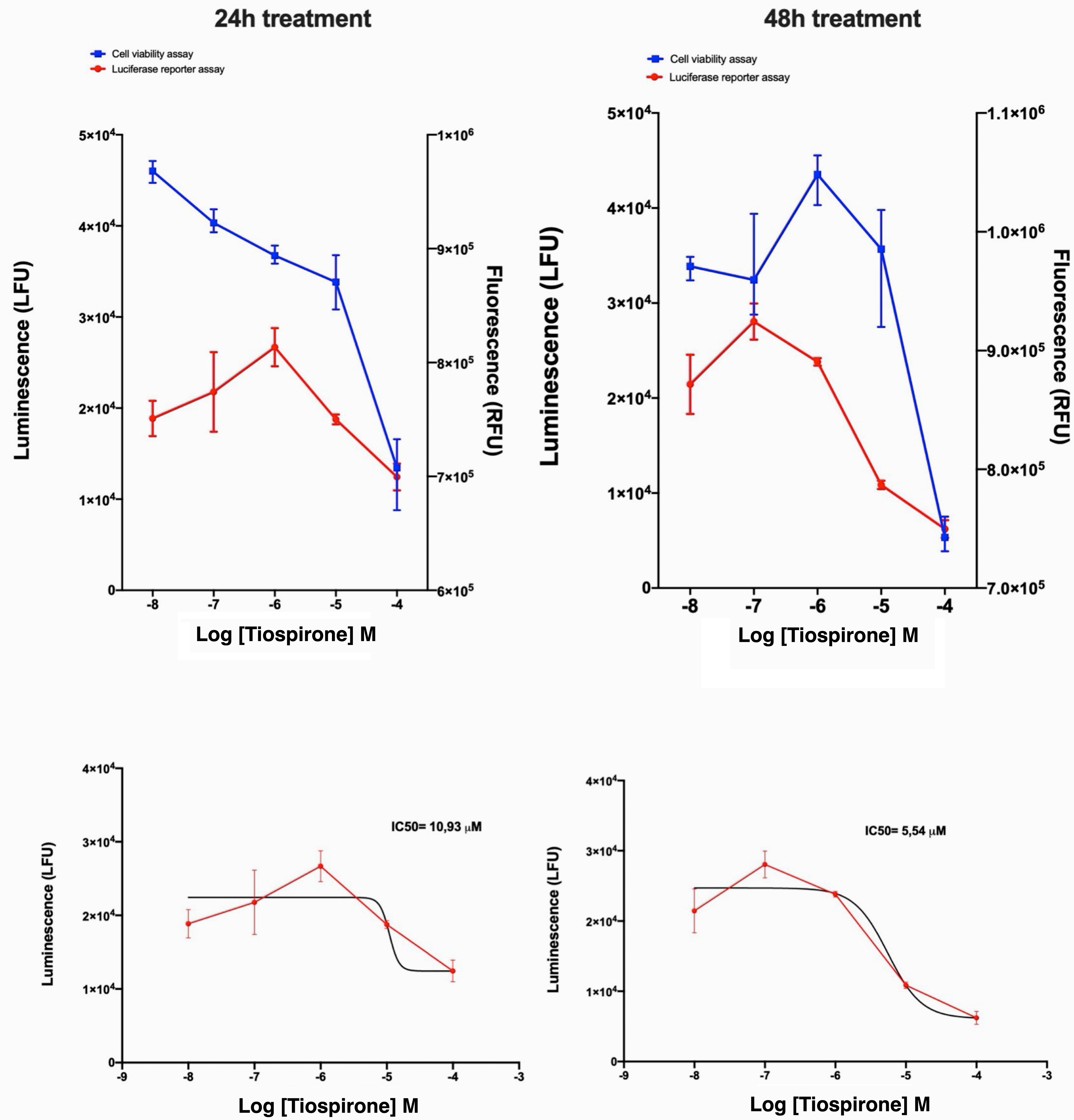

24h treatment
48h treatment
Cell viability assay
Luciferase reporter assay
Luminescence (LFU)
Fluorescence (RFU)
Log [Tiospirone] M
IC50= 10,93 μM
IC50= 5,54 μM
