# Supplementary material for: Experimental assessment of computationally nominated small molecules targeting the YAP–TEAD interface in Hippo pathway-altered mesothelioma cell models
Source: Front Oncol. 2026 Sep 14;16:1908974. doi: 10.3389/fonc.2026.1908974 (PMC13616692; doi:10.3389/fonc.2026.1908974)
Supplement: Supplementary file 2 [file Table1.docx]

**Supplementary Table 1. Compounds evaluated in the exploratory MCF7 TEAD-responsive reporter, with reporter IC₅₀ where fitted, viability-coupling status, and outcome.**

| **Compound** | **Assay(s)** | **Cell model** | **Reporter IC₅₀ (µM)** | **Reporter suppression viability-coupled?** | **Outcome** |
| --- | --- | --- | --- | --- | --- |
| Montelukast | TEAD reporter | MCF7 | 13.75 (24 h) / 15.91 (48 h) | Yes | Inactive (viability-coupled) |
| Tiospirone | TEAD reporter; Annexin V | MCF7 (reporter); NCI-H226 (apoptosis) | 10.93 (24 h) / 5.54 (48 h) | Yes | Inactive (viability-coupled) |
| Fisetin | TEAD reporter | MCF7 | 85.75 (24 h) / 35.72 (48 h) | Yes | Inactive (viability-coupled) |
| AK968/15360638 | TEAD reporter | MCF7 | 27.4 | Yes | Inactive (viability-coupled) |
| AN988/15131316 | TEAD reporter | MCF7 | 20.4 | Yes | Inactive (viability-coupled) |
| AK968/12973510 | TEAD reporter | MCF7 | 1.5 | Yes | Inactive (poor fit) |
| 0091-0033 | TEAD reporter | MCF7 | 12.2 | Yes | Inactive (poor fit) |
| 3201-0179 | TEAD reporter | MCF7 | 84.67 | Yes | Inactive (poor fit) |
| 8005-8632 | TEAD reporter | MCF7 | 96.65 | Yes | Inactive (poor fit) |
| 8004-0590 | TEAD reporter | MCF7 | 141.5 | Yes | Inactive (poor fit) |
| 8007-7516 | TEAD reporter | MCF7 | >100 | Yes | Inactive (viability-coupled) |
| Fluspirilene | TEAD reporter | MCF7 | No fit | No | Inactive |
| Altanserine | TEAD reporter | MCF7 | No fit | No | Inactive |
| A205/11187079 | TEAD reporter | MCF7 | No fit | No | Inactive |
| AG219/13129046 | TEAD reporter | MCF7 | No fit | n/a | Toxic (non-selective) |
| AG690/10485018 | TEAD reporter | MCF7 | No fit | n/a | Toxic (non-selective) |
| 3764-6801 | TEAD reporter | MCF7 | No fit | n/a | Toxic (non-selective) |
| 5586-0460 | TEAD reporter | MCF7 | No fit | n/a | Toxic (non-selective) |
| C878-0069 | TEAD reporter | MCF7 | No fit | n/a | Toxic (non-selective) |
| F550-4022 | TEAD reporter | MCF7 | No fit | n/a | Toxic (non-selective) |
| Y500-5709 | TEAD reporter | MCF7 | No fit | n/a | Toxic (non-selective) |
| Tomelukast | TEAD reporter | MCF7 | No fit | n/a | Toxic at 48 h |
| Zafirlukast | TEAD reporter | MCF7 | No fit | n/a | Toxic at 48 h |

CTG, CellTiter-Glo viability assay. The 18-compound SPECS library set was tested at a single timepoint; the leukotriene-class set (montelukast, tomelukast, fisetin, zafirlukast, tiospirone) was tested at 24 and 48 h. All reporter assays were performed in MCF7 cells in technical triplicate across a five-point concentration range (10 nM to 100 µM).
